# Examining the Correlation Between Depression and Social Behavior on Smartphones Through Usage Metadata: Empirical Study

**DOI:** 10.2196/19046

**Published:** 2021-01-06

**Authors:** Yameng Wang, Xiaotong Ren, Xiaoqian Liu, Tingshao Zhu

**Affiliations:** 1 CAS Key Laboratory of Behavioral Science Institute of Psychology Chinese Academy of Sciences Beijing China; 2 School of Computer Science and Technology University of Chinese Academy of Sciences Beijing China; 3 College of Foreign Languages Capital Normal University Beijing China; 4 Department of Psychology University of Chinese Academy of Sciences Beijing China

**Keywords:** depression, digital phenotyping, social behavior, smartphone usage, mobile sensing

## Abstract

**Background:**

As smartphone has been widely used, understanding how depression correlates with social behavior on smartphones can be beneficial for early diagnosis of depression. An enormous amount of research relied on self-report questionnaires, which is not objective. Only recently the increased availability of rich data about human behavior in digital space has provided new perspectives for the investigation of individual differences.

**Objective:**

The objective of this study was to explore depressed Chinese individuals’ social behavior in digital space through metadata collected via smartphones.

**Methods:**

A total of 120 participants were recruited to carry a smartphone with a metadata collection app (MobileSens). At the end of metadata collection, they were instructed to complete the Center for Epidemiological Studies-Depression Scale (CES-D). We then separated participants into nondepressed and depressed groups based on their scores on CES-D. From the metadata of smartphone usage, we extracted 44 features, including traditional social behaviors such as making calls and sending SMS text messages, and the usage of social apps (eg, WeChat and Sina Weibo, 2 popular social apps in China). The 2-way ANOVA (nondepressed vs depressed × male vs female) and multiple logistic regression analysis were conducted to investigate differences in social behaviors on smartphones among users.

**Results:**

The results found depressed users received less calls from contacts (all day: *F_1,116_*=3.995, *P*=.048, η_2_=0.033; afternoon: *F_1,116_*=5.278, *P*=.02, η_2_=0.044), and used social apps more frequently (all day: *F_1,116_*=6.801, *P*=.01, η_2_=0.055; evening: *F_1,116_*=6.902, *P*=.01, η_2_=0.056) than nondepressed ones. In the depressed group, females used Weibo more frequently than males (all day: *F_1,116_*=11.744, *P*=.001, η_2_=0.092; morning: *F_1,116_*=9.105, *P*=.003, η_2_=0.073; afternoon: *F_1,116_*=14.224, *P*<.001, η_2_=0.109; evening: *F_1,116_*=9.052, *P*=.003, η_2_=0.072). Moreover, usage of social apps in the evening emerged as a predictor of depressive symptoms for all participants (odds ratio [OR] 1.007, 95% CI 1.001-1.013; *P*=.02) and male (OR 1.013, 95% CI 1.003-1.022; *P*=.01), and usage of Weibo in the morning emerged as a predictor for female (OR 1.183, 95% CI 1.015-1.378; *P*=.03).

**Conclusions:**

This paper finds that there exists a certain correlation between depression and social behavior on smartphones. The result may be useful to improve social interaction for depressed individuals in the daily lives and may be insightful for early diagnosis of depression.

## Introduction

Depression is a mental disorder that is widespread in the world, with more than 300 million people affected [1]. Subthreshold depressive symptoms are far more common, causing significant impairment in people’s lives and putting them at risk for future mental health concerns [2]. Depression is not only associated with decreased quality of life [3], decreased work productivity [4], and physical illnesses such as cardiovascular problems [5] and Alzheimer disease [6], but also increases the mortality and suicide rate [7,8]. The World Health Organization estimates that depression will be the leading cause of disease burden by 2030 [9].

Although depression is treatable using different methods, including antidepressants and psychotherapy, only fewer than half of those eligible received treatment [10,11]. One of the main obstacles is the difficulty of diagnosing depression. For instance, guideline-concordant care for depressed patients seeking treatment is typically initiated by primary care physicians [12,13], whereas primary care physicians might fail to identify most patients with depressive symptoms [14,15]. Because early treatment and intervention are shown to be associated with a better prognosis [16], more efficient methods of identifying depression could significantly improve the delivery of services to those in need.

Currently, smartphone has led to an enormous increase in personal convenience and effectiveness, with more than 3 billion users worldwide [17]. Despite the many uses and advantages of smartphones, they also bring many negative effects on the mental health of individuals (eg, depression). There has been evidence that general and problematic smartphone usage commonly co-occur with depression. On the one hand, some studies found that depression can cause smartphone addiction, because depressed individuals take their smartphones as a coping method to deal with their depressive emotion [18,19]. On the other hand, some studies found that high smartphone usage is associated with subsequent stress, sleep diﬃculties, and depression [20,21]. Furthermore, some studies found a bidirectional relationship between smartphone usage and depression [22]. Specifically, depressed individuals may be driven to excessively use their smartphone to get rid of negative emotions, but this excessive smartphone usage consequently elicits more sleep problems, depression, irritability, and stress.

Communication and interpersonal interaction are among the most important functions of smartphones, and much research has been conducted on the relationship between depression and social behaviors on smartphones. While a smartphone is essential for communication and interpersonal interaction, it may make people less engaged with their real-life social environment [23-25]. Several studies found that depressed users have been reported to make fewer calls, but send more SMS text messages [26-28]. Kim et al [22] found that depressed users may rely on smartphones to alleviate their negative feelings and spend more time on communication, which in turn can deteriorate into problematic outcomes. Tamura et al [29] found that smartphone usage of 2 hours or more per day for social network services and online chats is associated with a higher risk of depression.

However, the majority of relevant studies measured smartphone usage using a self-report methodology. And self-reported smartphone usage did not correlate robustly with objectively measured smartphone usage [30,31]. Only recently, the increased availability of smartphone-based passive sensing data has provided new perspectives for the investigation of physical, individual differences, or mental health [32-34]. For instance, Saeb et al [35] found that both smartphone usage duration and frequency are positively associated with depression by analyzing daily life behavioral markers obtained using smartphone GPS and usage sensors. Using a smartphone-based self-monitoring system, Faurholt et al [36] found that depressive state in patients with bipolar disorder was related to more screen time, while manic state was related to frequent smartphone usage. Furthermore, it has been demonstrated that behavior data collected via smartphone can be used for monitoring individual depressive states [37], detecting social rhythms in bipolar disorder [38], and mobile intervention for depression [39]. Therefore, we aim to conduct research on depressed users’ social behavior in digital space through metadata collected via smartphones.

To gain deeper insights, we include a broad range of behaviors based on previous studies which have reported on behavioral manifestations of depression in various types of behavior. For example, traditional communication behaviors (eg, making calls, sending SMS text messages) have been shown to be associated with depressive symptoms in many studies [23,27,28]. Moreover, as Baker et al [40] reviewed, there is a complex relationship between online social networking and depressive symptoms. Given that 99.1% of internet users are mobile internet users in China [41], usage of social apps on smartphones may be also associated with depression.

In addition, gender differences exist not only in depression but also in smartphone usage. Specifically, males with depression show impairment at lower symptom levels than females [42], and report consistently fewer symptoms than females [43]. As for gender differences in smartphone usage, several studies found that female users’ smartphone usage is typically related to sociability, interpersonal relationships, and the creation of new relationships [44,45], but for male users, smartphone usage is simultaneously based on SMS text messages and voice conversations [46,47]. Furthermore, virtually all the studies indicate that females have higher levels of dependence and problematic usage than males [48,49]. Therefore, gender differences may also exist in the social behaviors on smartphones of depressed users.

By analyzing smartphone usage, this paper aims to examine the correlation between depression and social behavior on smartphones through metadata collected via smartphones. Specifically, we hypothesized that nondepressed and depressed users may have differences in social behaviors on smartphones, and there are gender differences in social behavior among depressed users.

## Methods

### Study Procedure

In this study, a 2-step procedure was conducted: (1) data collection and (2) data analysis. In order to collect metadata of smartphone usage automatically, we developed an Android app named MobileSens [50,51]. MobileSens consists of 2 modules, one is for collecting metadata of smartphone usage, and the other is for filling the questionnaire, which can get smartphone usage data and the corresponding questionnaire results at the same time.

### Data Collection

#### Participants

We recruited participants in Beijing, China, who own Android smartphones and also use smartphones in daily life by advertising on social networks. During this study, all participants were instructed to install MobileSens on their smartphones, and use it for more than 1 month. Given psychological measurement scales are composed of retrospective questions, participants were asked to fill out the corresponding questionnaires on the last day of metadata collection to ensure consistency between metadata and participants’ psychological state. To encourage participants to take part in our study, we offered them a monetary reward depending on the number of days the smartphone usage data was uploaded. In particular, if participants finished uploading 30 days’ data and completed the questionnaires, we rewarded them with RMB 200 (US $30). However, if participants dropped out of the study after finishing questionnaires and uploading data for less than 30 days, we provided experimental rewards based on the number of days the data were uploaded. This study was approved by the Review Board of Institute of Psychology, Chinese Academy of Sciences, H09036. Signed informed consent was obtained from each participant. Finally, we acquired data from a total of 120 participants (73 males, 47 females) with a mean age of 23.57 (SD 3.09).

#### Metadata of Smartphone Usage

MobileSens ran in the background to collect designated metadata. After participants installed MobileSens into their smartphone, most of their interactions with the smartphone were recorded and no sensitive personal data, such as the specific content of the SMS text messages, were collected. This app sensed a total of 15 categories of smartphone interaction events, as listed in Table 1. All the data would be uploaded via the internet to a remote central data server.

**Table 1:** Details on smartphone log data.

Category	Record content
App activity log	App creation/resume/launch/stop/exit
App package log	App package uninstallation/installation/replacement/change/data cleanup/restart
Call log	Number, contact name, and direction of call
Camera log	Use of camera button
Contact log	Contacts addition/deletion
Date change log	Change system date and time
GPS log	User’s locale, altitude, latitude, longitude, and direction of movement
Headset log	Plug in/off headset
Location changed log	Change location
Power connected log	Connect/disconnect the power
Power log	Power on/off
Screen log	Screen on/off
Service app log	Service app creation/resume/launch/stop/exit
SMS text message log	Number, contact name, and direction of SMS text message
Wallpaper log	Change wallpaper

#### Questionnaires

On the last day of the study, each participant was required to complete an online assessment using MobileSens, consisting of a demographic questionnaire and a depression measurement scale. Depressive symptoms (minor depression) were evaluated using the Center for Epidemiological Studies-Depression Scale (CES-D) [52], which is widely used to measure depressive symptoms severity in the general population and is applicable to different ethnic contexts [53]. The CES-D measures the frequency of 20 depressive symptoms over the past week on a 4-point Likert scale. The total score may range from 0 to 60, with higher scores indicating higher levels of depression. In this study the Chinese version of questionnaire was implemented, and their validity and reliability have been proved previously [54].

### Data Analysis

#### Feature Extraction

Among the metadata of smartphone usage sensed by MobileSens, the analyses of this work focused on call log, SMS text message log, and app activity log. These metadata are directly or, in some cases, indirectly related to individuals’ social behavior on smartphones. For instance, call log and SMS text message log are the direct output of individuals’ communication behavior; app activity log belonging to social apps can reflect individuals’ online social activities, although social apps also have entertainment and information acquisition functions. Specifically, we designed 2 categories of social behaviors on metadata:

##### Traditional Communication Behavior

We mainly focused on the frequency of usage of calls and SMS text messages. In particular, we designed 4 kinds of behaviors related to traditional communication behavior from the metadata (making calls, receiving calls, sending SMS text messages, and receiving SMS text messages). In order to analyze the communication behavior between individuals and acquaintances (eg, contacts on smartphones), we designed another 4 kinds of communication behaviors that occur between contacts, which included making calls to contacts, receiving calls from contacts, sending SMS text messages to contacts, and receiving SMS text messages from contacts. Finally, we obtained a total of 8 behaviors.

##### Usage of Social Apps

We analyzed the frequency of usage of social apps from the following 2 aspects. First, we analyzed the frequency of usage of all social apps. Specifically, using a web crawler, we crawled an app classification framework from the Wandoujia app store (one of the most popular Android app stores in China), and got a list of all the social apps. Then, we extracted from metadata the usage records of all social apps for social apps’ usage behavior. Second, we analyzed the frequency of usage of WeChat and Sina Weibo, 2 popular social apps in China, respectively. Finally, we designed a total of 3 behaviors.

After designing the above 2 categories of social behaviors, we extracted features from each category of behavior. Specifically, after calculating the frequency of all behaviors per day for each participant, we used the mean of the frequency of these behaviors as the feature of social behavior on smartphone all day (0:00 to 24:00). In addition, social activity represents the most important factor disrupting circadian rhythms after alternation of light/dark cycle [55], meaning that individuals’ social behavior may differ between day and night. Moreover, circadian rhythms have repeatedly been linked to individual depression [56,57]. Thus, it may make sense to analyze individuals’ social behaviors on smartphones during the day and at night. Further, some studies have found that many people with depression show a regular daily pattern of symptoms, usually with more severe symptoms in the morning [58,59]. To explore this difference, after dividing a day into day and night, we divided the day into morning and afternoon (morning: 6:00 to 12:00; afternoon: 12:00 to 18:00; evening: 18:00 to 6: 00). And then we calculated the average frequency of smartphone social behavior in different periods as the final features. Finally, we extracted a total of (8 + 3) × (1 + 3) = 44 behavior features.

#### Depressive Symptoms

To examine differences in social behaviors on smartphones between depressed and nondepressed users, we divided participants into 2 groups based on their scores on CES-D. The score of 16 has been traditionally used as a cutoff point for determining whether a person has symptoms of depression not only in Western countries [60], but also in China [54]. In this study, the cutoff point of 16 was used to distinguish individuals considered to be depressive from those classified as nondepressed.

### Statistical Analysis

Descriptive statistics were computed as the mean and SD for continuous variables and absolute frequencies and percentage for categorical variables. The chi-square test and independent *t*-test were used to compare demographics between 2 groups. The 2-way ANOVA of depressive symptom (nondepressed group vs depressed group) × gender (male vs female) was conducted to examine the differences in social behaviors. Multiple logistic regression analysis with dichotomous depressive symptoms (nondepressed vs depressed) as dependent variables was used to find predictors of depressive symptoms. In this analysis, variable selection was performed using stepwise forward selection, subsequently including one by one the variables that were not statistically significant (α=.05). To improve the chances of retaining meaningful predictor variables, demographics that differed between the groups at *P*<.1, and behavior features that differed in the main effect of depressive symptoms in 2-way ANOVA at *P*<.1 were analyzed as independent variables via multiple logistic regression analysis [61]. For further data exploration of gender differences, multiple logistic regression models adjusted for gender were conducted. All statistical tests were performed using SPSS version 23 for Windows (IBM). The level of significance was set at .05.

## Results

### Demographics and Questionnaire Scores of CES-D

Of the 120 participants who formed the study sample, 71 participants were divided into the nondepressed group and 49 participants were divided into the depressed group. Demographics of participants are summarized in Table 2. The results of the chi-square test and independent *t*-test on demographics between the nondepressed and depressed groups showed no significance (gender: *P*=.76; education: *P*=.34; living place: *P*=.99; occupation: *P*=.75; marital status: *P*=.36; and age: *P*=.31).

**Table 2:** Demographics of participant versus depressive symptoms.

Characteristic		Nondepressed group (N=71)	Depressed group (N=49)	χ^2^ (*df*) or *t* (*df*)	*P*-value
**Gender, n (%)**				0.10 (1)	.76^a^
	Male	27 (38)	20 (41)		
	Female	44 (62)	29 (59)		
**Education, n (%)**				2.16 (2)	.34^a^
	Below university diploma	3 (4)	0 (0)		
	University diploma	28 (39)	21 (43)		
	Above university diploma	40 (56)	28 (57)		
**Living place, n (%)**				0.02 (2)	.99^a^
	City	37 (52)	26 (53)		
	Town	13 (18)	9 (18)		
	Country	21 (30)	14 (29)		
**Occupation, n (%)**				0.10 (1)	.75^a^
	Student	64 (90)	45 (92)		
	Others	7 (10)	4 (8)		
**Marital status, n (%)**				0.83 (1)	.36^a^
	Single/windowed	32 (45)	18 (37)		
	Married/in a relationship	39 (55)	31 (63)		
Age, mean (SD)		23.34 (2.74)	23.92 (3.54)	1.01 (118)	.31^b^

^a^Chi-square test.

^b^Independent *t*-test.

### Differences in Social Behaviors on Smartphone

To examine differences in social behavior features between the nondepressed and depressed groups, we conducted 2-way ANOVA of depressive symptom (nondepressed group vs depressed group) × gender (male vs female). All social behavior features with significant results are shown in Table 3.

For “usage of traditional communication functions,” 2 behavioral features had significant differences. Specifically, in terms of “receiving calls from contacts all day,” the main effect of depressive symptom was significant (*F*_1,116_=3.995, *P*=.048,η^2^=0.033). The frequency of receiving calls from contacts all day was significantly higher in the nondepressed group than in the depressed group (*P*=.048). By contrast, the main effect of gender and the interaction effect of depressive symptom and gender were not significant (*F*_1,116_=0.005, *P*=.94, η^2^=0.000; *F*_1,116_=0.010, *P*=.92, η^2^=0.000). As for “receiving calls from contacts in the afternoon,” the main effect of depressive symptom was significant (*F*_1,116_=5.278, *P*=.02, η^2^=0.044). The frequency of receiving calls from contacts in the afternoon was significantly higher in the nondepressed group than in the depressed group (*P*=.02). However, the main effect of gender and the interaction effect of depressive symptom and gender were not significant (*F*_1,116_=0.004, *P*=.95, η^2^=0.000; *F*_1,116_=0.006, *P*=.94, η^2^=0.000).

In “usage of social apps,” 6 behavioral features had significant results. Specifically, in terms of “usage of social apps all day,” the main effect of depressive symptom was significant (*F*_1,116_=6.801, *P*=.01, η^2^=0.055). The frequency of usage of social apps was significantly higher in the depressed group than in the nondepressed group (*P*=.01). By contrast, the main effect of gender and the interaction effect of depressive symptom and gender were not significant (*F*_1,116_=0.675, *P*=.41, η^2^=0.006; *F*_1,116_=1.654, *P*=.20, η^2^=0.014). As for “usage of social apps in the evening,” the main effect of depressive symptom was significant (*F*_1,116_=6.902, *P*=.01, η^2^=0.056). People in the depressed group used social apps more frequently than people in the nondepressed group in the evening. However, the main effect of gender and the interaction effect of depressive symptom and gender were not significant (*F*_1,116_=0.095, *P*=.76, η^2^=0.001; *F*_1,116_=1.964, *P*=.16, η^2^=0.017).

**Table 3:** Differences in smartphone usage among participants with different depressive symptoms.^a^

Behavioral feature		Nondepressed group		Depressed group		*F*_group_ (*df*)	*P*_group_ value	*F*_gender_ (*df*)	*P*_gender_ value	*F*_interaction_ (*df*)	*P*_interaction_ value
		Male	Female	Male	Female						
**Traditional communication behavior, mean (SD)**											
	Receiving calls from contacts all day	0.49 (1.38)	0.48 (1.61)	0.05 (0.20)	0.09 (0.40)	3.995 (1, 116)	.048	0.005 (1, 116)	.94	0.010 (1, 116)	.92
	Receiving calls from contacts in the afternoon	0.20 (0.53)	0.20 (0.64)	0.01 (0.02)	0.02 (0.07)	5.278 (1, 116)	.02	0.004 (1, 116)	.95	0.006 (1, 116)	.94
**Usage of social apps, mean (SD)**											
	Usage of social apps all day	62.34 (114.24)	73.84 (107.88)	158.72 (172.41)	105.56 (120.28)	6.801 (1, 116)	.01	0.675 (1, 116)	.41	1.654 (1, 116)	.20
	Usage of social apps in the evening	26.30 (40.08)	42.61 (69.62)	86.42 (115.56)	60.90 (88.05)	6.902 (1, 116)	.01	0.095 (1, 116)	.76	1.964 (1, 116)	.16
	Usage of Weibo all day	6.64 (14.79)	6.31 (14.79)	6.34 (13.08)	25.49 (34.78)	6.689 (1, 116)	.01	6.642 (1, 116)	.01	7.118 (1, 116)	.009
	Usage of Weibo in the morning	1.46 (3.27)	1.23 (2.90)	1.94 (4.40)	6.11 (8.58)	8.838 (1, 116)	.004	4.765 (1, 116)	.03	5.932 (1, 116)	.02
	Usage of Weibo in the afternoon	1.88 (4.59)	2.11 (5.57)	1.58 (3.17)	8.19 (10.81)	6.370 (1, 116)	.01	8.918 (1, 116)	.003	7.764 (1, 116)	.006
	Usage of Weibo in the evening	3.30 (7.40)	2.97 (6.73)	2.82 (5.97)	11.19 (17.93)	4.531 (1, 116)	.04	4.889 (1, 116)	.03	5.731 (1, 116)	.02

^a^Data for descriptive statistics are the mean (SD) of the frequency of social behavior on smartphone. *F* values of 2-way ANOVA are represented by *F*_group_, *F*_gender_, and *F*_interaction_. *P* values of 2-way ANOVA are represented by *P*_group_ value, *P*_gender_ value, and *P*_interaction_ value.

**Figure 1:**
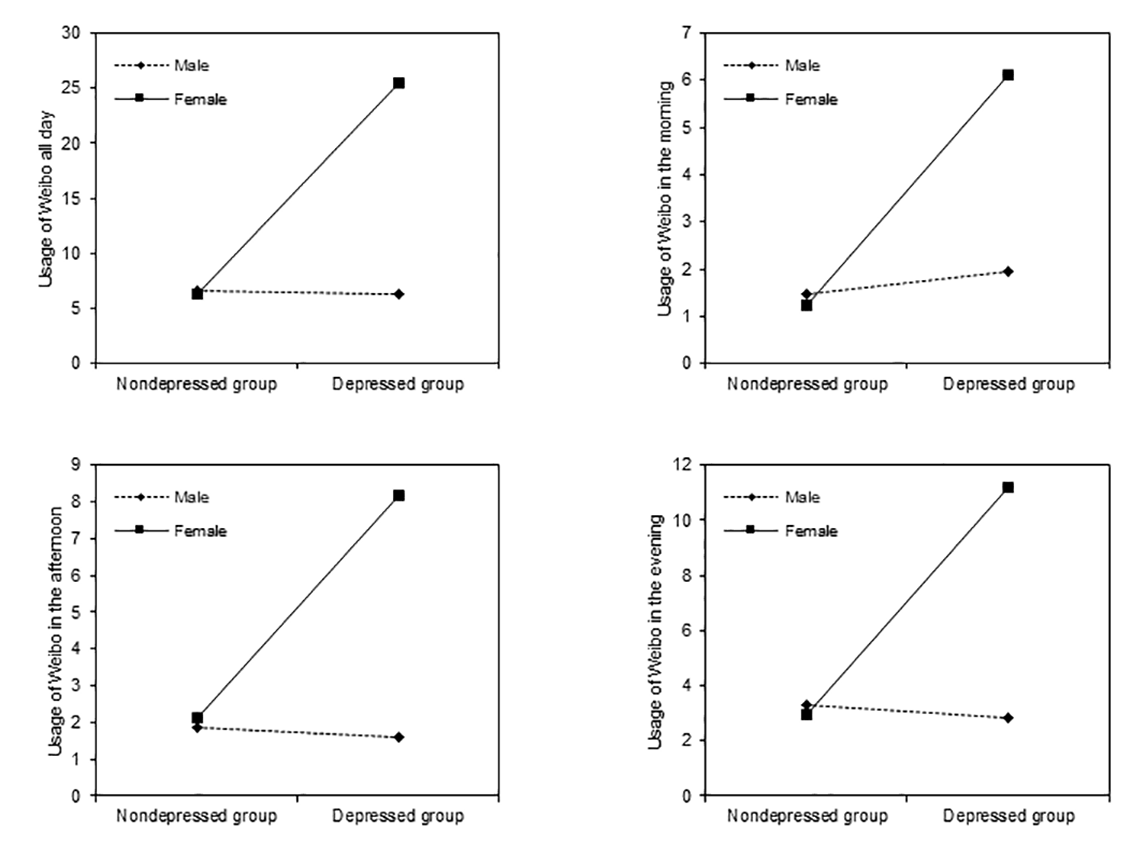
Interactions between depressive symptom and gender on usage of Weibo in different periods.

In addition, Weibo was the only app with significant results among the 2 popular social apps. Under “usage of Weibo all day,” the main effect of depressive symptom, the main effect of gender, and the interaction effect of depressive symptom and gender were significant (*F*_1,116_=6.689, *P*=.01, η^2^=0.055; *F*_1,116_=6.642, *P*=.01, η^2^=0.054; *F*_1,116_=7.118, *P*=.009, η^2^=0.058, respectively). Simple effect analyses showed that in the depressed group, females used Weibo more frequently all day than males (*F*_1,116_=11.744, *P*=.001, η^2^=0.092; Figure 1). However, in the nondepressed group, there were no significant differences (*F*_1,116_=0.005, *P*=.94, η^2^=0.000). As for “usage of Weibo in the morning,” the main effect of depressive symptom, the main effect of gender, and the interaction effect of depressive symptom and gender were significant (*F*_1,116_=8.838, *P*=.004, η^2^=0.071; *F*_1,116_=4.765, *P*=.03, η^2^=0.039; *F*_1,116_=5.932, *P*=.02, η^2^=0.049, respectively). Simple effect analyses showed that in the depressed group, the frequency of usage of Weibo among females in the morning was significantly higher than that of males (*F*_1,116_=9.105, *P*=.003, η^2^=0.073; Figure 1). However, in the nondepressed group, no significant gender differences existed (*F*_1,116_=0.039, *P*=.85, η^2^=0.000).

Concerning “usage of Weibo in the afternoon,” the main effect of depressive symptom, the main effect of gender, and the interaction effect of depressive symptom and gender were significant (*F*_1,116_=6.370, *P*=.01, η^2^=0.052; *F*_1,116_=8.918, *P*=.003, η^2^=0.071; *F*_1,116_=7.764, *P*=.006, η^2^=0.063, respectively). Simple effect analyses showed that in the depressed group, females used Weibo more frequently in the afternoon than males (*F*_1,116_=14.224, *P*<.001, η^2^=0.109; Figure 1). However, in the nondepressed group, no significant gender differences existed (*F*_1,116_=0.024, *P*=.88, η^2^=0.000). In terms of “usage of Weibo in the evening,” the main effect of gender and the interaction effect of depressive symptom and gender were not significant (*F*_1,116_=4.531, *P*=.04, η^2^=0.038; *F*_1,116_=4.889, *P*=.03, η^2^=0.040; *F*_1,116_=5.731, *P*=.02, η^2^=0.047, respectively). Simple effect analyses showed that in the depressed group, females used Weibo more frequently than males in the evening (*F*_1,116_=9.052, *P*=.003, η^2^=0.072; Figure 1). However, in the nondepressed group, no significant gender differences existed (*F*_1,116_=.020, *P*=.89, η^2^=0.000).

### Predictors of Depressive Symptoms

The following 11 variables were entered into the multiple logistic regression model: “receiving calls from contacts all day,” “receiving calls from contacts in the afternoon,” “receiving calls from contacts in the evening,” “usage of social apps all day,” “usage of social apps in the morning,” “usage of social apps in the evening,” “usage of Weibo all day,” “usage of Weibo in the morning,” “usage of Weibo in the afternoon,” “usage of Weibo in the evening,” and “usage of WeChat in the evening.” Of the 11 variables, “usage of social apps in the evening” remained a significant independent predictor (*P*=.02) of depressive symptoms in the model established on the whole sample (Table 4). In addition, “usage of social apps in the evening” emerged as a significant factor for male (*P*=.01), and “usage of Weibo in the morning” emerged as a significant factor for female (*P*=.03).

**Table 4:** Multiple logistic models of depressive symptoms.

Behavioral feature		Odds ratio (95% CI)	*P* value
**Total**			
	Usage of Weibo in the morning	1.089 (0.996-1.191)	.06
	Usage of social apps in the evening	1.007 (1.001-1.013)	.02
	Receiving calls from contacts in the afternoon	0.032 (0.000-2.705)	.13
**Male**			
	Usage of social apps in the evening	1.013 (1.003-1.022)	.01
**Female**			
	Usage of Weibo in the morning	1.183 (1.015-1.378)	.03

## Discussion

### Principal Findings

Our results supported the hypothesis that nondepressed and depressed users have differences in social behaviors on smartphones, and there were gender differences in social behavior among depressed users. Specifically, we used MobileSens to obtain metadata of smartphone usage on 120 participants, and extracted social behavior features to investigate the social behavior of nondepressed and depressed users. The results found depressed users received less calls from contacts (all day, in the afternoon) and used social apps more frequently (all day, in the evening) than nondepressed users. In the depressed group, females used Weibo more frequently than males (all day, in the morning, in the afternoon, in the evening).

### Traditional Communication Behavior of Depressed Users

The results show that depressed users receive fewer calls from contacts throughout the day than nondepressed users. Consistent with our results, a cross-sectional study involving 6105 adults aged 18-84 years found that low frequency of contacts with friends and parents was a significant factor associated with depression [62]. In particular, social interactions with strong ties (eg, family, friends) play an important role in satisfying people’s need for social connectedness [63-66]. Furthermore, meta-analyses have also shown that interventions for depression by addressing social relationships, including couples therapy and peer support, may be effective in reducing depressive symptoms [67,68]. Therefore, it is plausible that social interactions with strong ties via calls can satisfy psychological needs essential for reduction of depression and human flourishing. In addition, the result can be supplemented by one of the major assumptions of the behavioral theory of depression, namely, that a low rate of response-contingent positive reinforcement acts as an eliciting stimulus for depression [69-71]. Response-contingent positive reinforcement is defined as pleasurable or positive outcomes that follow an individual’s behavior within his or her environment and increase the likelihood of those behaviors. Under such an assumption, social interactions with friends through calls could be perceived as a positive response contingent, whereas lack of social interaction with friends or family will increase depression.

Notably, only in the afternoon did the 2 groups differ significantly in the frequency of calls received from contacts over all periods (*P*=.02). Previous studies have found a circadian rhythm of positive affect in healthy individuals, peaking in the afternoon [72,73]. Moreover, this positive affect was positively related to the number of interactions in which participants engaged, and the amount of time spent engaged in social contact [74]. According to the social enhancement model [75], those who already have good mental state enjoy more benefits from social interactions, while problems are only compounded for those who have psychological deficits. Thus, people who are already in good mental states have more willingness to engage in social interactions, which may encourage others to call them. However, unlike healthy individuals, the diurnal variation of positive affect in depressed patients showed an inverse-U shape with a steeper overall increase from morning lows to evening highs [76]. Thus, it is not surprising that difference exists in the number of receiving calls in the afternoon between the 2 groups.

### Usage of Social Apps of Depressed Users

We found that depressed users use social apps throughout the day more frequently than nondepressed ones. There might be several reasons. First, depressed people expect that interpersonal interaction via the internet can alleviate their psychological problems, because they believe that it is less risky and easier to get support and build relationships online than face to face [77,78]. Given that 99.1% of internet users are mobile internet users in China [41], the positive association between depression and internet overuse would be replicated for a smartphone. Second, deﬁciency in self-regulation is a common manifestation among those with depression [79], which makes it difﬁcult for them to maintain a healthy level and amount of smartphone use. Therefore, depressed users use social apps more frequently than nondepressed ones.

As for “usage of social apps in the evening,” we found that it is a predictor for depression on both the overall data (all participants) and male data (male participants), and the usage frequency of depressed users is higher than nondepressed ones. Previous studies have found that depression symptoms tend to be relatively more active during the evening and night [59,80-82], which may exacerbate the usage of social apps. Additionally, people prefer to spend time on social media rather than face-to-face activities [83-85], but the quality of communication over social media has been highlighted as a potential limiting factor in building strong, emotionally intense relationships [86-88]. Furthermore, frequent smartphone usage can make people vulnerable to negative outcomes, such as interpersonal isolation [89]. Therefore, the usage of social apps not only fails to lessen their negative emotions, but also actually worsens these outcomes.

In the usage of top 2 social apps, at any time, the depressed ones used Weibo more frequently than the nondepressed ones. In the depressed group, females used Weibo more frequently than males. As we discussed above, the depressed ones use social apps more than the nondepressed ones, so this conclusion applies equally to Weibo. What deserves our attention is the gender differences in Weibo usage in the depressed group. Similar to Twitter, but unlike WeChat, Weibo focuses on sharing of opinions and information exchange rather than on social interaction [90], and offers some anonymity in online communication [91]. A previous study found that females are more likely to report Weibo usage for communicating with peers, passing time, and entertainment, whereas male users are more likely than females to report Weibo use for social compensation and social identity gratifications [92]. So depressed females are more likely to overuse Weibo than depressed males. Given a worsening of symptoms in the morning hours and early morning waking are characteristics of depression [58], depressed females may try to eliminate psychological problems by using Weibo. This could explain the usage of Weibo in the morning, which could serve as a predictor of depression in females.

### Limitations and Future Work

One limitation of this study is that we only use questionnaire-based scales to measure depression. Although the validity of the questionnaires as a screening tool in accessing depression severity has been well proven in the literature, the questionnaire score itself cannot be used as diagnostic. Second, the sample in this study was composed of healthy individuals rather than clinical patients, which means that there were few “real” patients with depression disorders in our sample. So our results are applicable to individuals with minor depression, and their applicability to patients with confirmed moderate to severe depression has not been verified. Moreover, given the convenience sampling method of this study and the majority of participants were students, the extent to which sampling bias affected the results cannot be determined. Therefore, replication and extension of this research using larger, more representative samples are desirable in future work.

Although the results found that depressed users used social apps more frequently than nondepressed ones, the psychological mechanism behind it is still unclear. For instance, because deﬁciency in self-regulation is a common manifestation among those with depression [79], future work could explore the mediating effect of self-regulation in the relationship between depression and social behavior on smartphones. Additionally, our results showed that compared with nondepressed users, depressed users received fewer calls from acquaintances. Future studies may need to pay closer attention to whether it is the reason that depressed users use social apps frequently. Furthermore, our result found gender differences in some social behaviors among depressed users (eg, the usage of Weibo), which suggests that social-based features should be treated as a personalized feature that should be assessed in a within-subject analysis in future studies.

### Conclusions

In summary, by analyzing metadata of smartphone usage acquired by MobileSens, we found that relationships exist between depression and social behavior on smartphones, which means we may be able to implement an early diagnosis system of depression through smartphone usage. Besides, the results of this study provide useful suggestions to those who are depressive in daily lives.

## Abbreviations

CES-D: The Center for Epidemiological Studies-Depression Scale
OR: odds ratio
